# RNA-Seq analysis and transcriptome assembly for blackberry (*Rubus* sp. Var. Lochness) fruit

**DOI:** 10.1186/s12864-014-1198-1

**Published:** 2015-01-22

**Authors:** Daniel Garcia-Seco, Yang Zhang, Francisco J Gutierrez-Mañero, Cathie Martin, Beatriz Ramos-Solano

**Affiliations:** Facultad de Farmacia, Universidad CEU San Pablo, Ctra. Boadilla del Monte km 5.3, Boadilla del Monte, 28668 Madrid Spain; John Innes Center, Norwich Research Park, Norwich, NR4 7UH UK

**Keywords:** Blackberry, RNA-seq, *de novo*, Transcriptome, SNP, Alternative splicing, Chimera

## Abstract

**Background:**

There is an increasing interest in berries, especially blackberries in the diet, because of recent reports of their health benefits due to their high content of flavonoids. A broad range of genomic tools are available for other *Rosaceae* species but these tools are still lacking in the *Rubus* genus, thus limiting gene discovery and the breeding of improved varieties.

**Results:**

*De novo* RNA-seq of ripe blackberries grown under field conditions was performed using Illumina Hiseq 2000. Almost 9 billion nucleotide bases were sequenced in total. Following assembly, 42,062 consensus sequences were detected. For functional annotation, 33,040 (NR), 32,762 (NT), 21,932 (Swiss-Prot), 20,134 (KEGG), 13,676 (COG), 24,168 (GO) consensus sequences were annotated using different databases; in total 34,552 annotated sequences were identified. For protein prediction analysis, the number of coding DNA sequences (CDS) that mapped to the protein database was 32,540. Non redundant (NR), annotation showed that 25,418 genes (73.5%) has the highest similarity with *Fragaria vesca* subspecies *vesca*. Reanalysis was undertaken by aligning the reads with this reference genome for a deeper analysis of the transcriptome. We demonstrated that *de novo* assembly, using Trinity and later annotation with Blast using different databases, were complementary to alignment to the reference sequence using SOAPaligner/SOAP2. The *Fragaria* reference genome belongs to a species in the same family as blackberry (*Rosaceae*) but to a different genus. Since blackberries are tetraploids, the possibility of artefactual gene chimeras resulting from mis-assembly was tested with one of the genes sequenced by RNAseq, *Chalcone Synthase* (*CHS*). cDNAs encoding this protein were cloned and sequenced. Primers designed to the assembled sequences accurately distinguished different contigs, at least for chalcone synthase genes.

**Conclusions:**

We prepared and analysed transcriptome data from ripe blackberries, for which prior genomic information was limited. This new sequence information will improve the knowledge of this important and healthy fruit, providing an invaluable new tool for biological research.

**Electronic supplementary material:**

The online version of this article (doi:10.1186/s12864-014-1198-1) contains supplementary material, which is available to authorized users.

## Background

The *Rosaceae* comprise a moderately large family, with an estimated 85 genera and approximately 2,000 sexual species [1], that include several economically and nutritionally important crops cultivated worldwide, such as apples (*Malus domestica* L.), plums (*Prunus domestica* L.), pears (*Pyrus communis* L.), cherries (*Prunus avium* L.) and peaches (*Prunus persica* L. Batsch.).

Among the *Rosaceae* are blackberries (*Rubus* spp), with growing market interest due to recent discoveries about the beneficial effects of polyphenols for human health [2,3]. Therefore, increasing polyphenol levels will likely result in healthier fruits. However, despite the agricultural and biological importance of *Rubus*, knowledge of the genetics and genome is limited. So far, genomic efforts have focused largely on other *Rosaceae*, including apple (*Malus x domestica*), strawberry (*Fragaria vesca*), peach (*Prunus persica*) and pear (*Pyrus communis*) for which whole genome sequencing has been completed by combining traditional Sanger, Roche and next-generation Illumina GA sequencing technologies [4-7]. Many genomic resources for these *Rosaceae* species are available on the Genome Database for *Rosaceae* website [8].

The genus *Rubus* is classified into 12 subgenera, and at the same time is divided into sections or subseries. Economically, the most important subgenus is *Rubus*, with more than 130 species [9-11]. During the domestication of blackberries, parentals with desirable traits such as large, sweet fruits, thornlessness and high yield have been hybridised, resulting in complex hybrids comprising elite commercial lines.

The variety *Rubus* spp. var. Lochness is a high yielding tetraploid (4n = 28) blackberry, and one of the most widely cultivated varieties. Its origin is Scottish, with parents SCRI 74126RA8 x SCRI 75131D2 hybrid complex, obtained in 1998 in Invergowrie, Scotland, by the Scottish Crop Research Institute [12]. It is a mixture of races, among which the most prominent are “Comanche” and “Merton Thornless”. The parents came from Scotland and North America, and include European species; *Rubus ulmifolius*, *R. trivialis, R. strigosus,* and *R. armeniacus*. Commercially, the term often used is *fruticosus*, although from a botanical point of view, it is more accurately described as *Rubus* spp. var. Loch Ness.

The lack of dense genetic maps, large high-throughput marker collections, and suitable mapping populations has limited gene isolation and breeding in blackberry. Several *Rubus* genes have been reported, mainly related to fruit quality, especially genes encoding the enzymes of phenylpropanoid metabolism, and resistance to diseases [13-19], but most of these are derived from raspberry (*Rubus idaeus*) and none of them have been cloned and characterized molecularly in blackberry.

Currently, there are more than 74.2 million ESTs in the NCBI public collection [20]. However, less than 3,200 EST sequences are available collectively for all the *Rubus* species, and approximately 540,000 for all the species in the *Rosaceae* family, compared to more than 1.8 and 2 million ESTs available for *Arabidopsis* and *Zea mays*, respectively.

RNA-Seq is a powerful tool for transcriptome analysis and uses deep-sequencing technologies to produce millions of short cDNA reads. The resulting reads are either aligned to a reference genome or reference transcripts, or assembled de novo (without the genomic sequence) to produce a genome-scale transcription map that consists of both the transcript structure and level of expression for each gene at any particular developmental stage [21-25].

Here, we describe the generation of almost 9 billion nt bases using Illumina RNA-Seq technology, and the detection of 42,062 consensus sequences. These sequences are functionally annotated and represent the first *Rubus* sp. transcriptome. We show that *de novo* assembly using Trinity [26] and a later annotation with BLAST interrogation of different databases is complementary to alignment to a reference sequence with SOAPaligner/SOAP2 [27], using as a reference, the genome sequence a species of the same family (*Rosaceae*), but a different genus, *Fragaria*. Access to the sequence of the ripe blackberry transcriptome will accelerate genetic analysis and breeding of this crop and facilitate attempts to improve fruit quality based on secondary metabolite accumulation, and improved field performance within more sustainable production.

We have demonstrated the suitability of short-read sequencing for de novo assembly and annotation of genes without prior genome information, as well as its reliability and complementarity by alignment with sequence from a close, but distinct, species. Our results will facilitate the discovery of new functional genes in *Rubus* sp. Based on this background, the aim of this study was founded on i) the importance of the transcriptome to improvement of fruit quality dependent on secondary metabolite content, which will be useful for breeders and biotechnologists, and ii) comparison of bioinformatic approaches to study a new transcriptome, with little or no pre-existing information of its genome.

## Results and discussion

### Sample preparation and Illumina sequencing

RNA-seq was performed on ripe fruit of *Rubus* sp var Lochness to gather information about genes expressed at the time and place most important to breeders of blackberry. Traits such as colour (anthocyanin biosynthesis), sweetness (sugar metabolism) and healthfulness (polyphenol metabolism) are determined by metabolic pathways active in ripe fruit. Total RNA of two independent samples, Ripe Fruit1 (RF1) and Ripe fruit2 (RF2) were isolated from ripe fruits to characterize the *Rubus* sp. transcriptome and enhance sequence coverage. After cleaning and quality checks, two independent rounds of Illumina sequencing (RF1 and RF2) generated 44,166,280 and 45,562,458 clean reads in total, encompassing 4,416,628,000 and 4,556,245,800 total nucleotides (nt) respectively (Table 1). These data sets are available in the EBI database (accession number: PRJEB6680).

**Table 1:** **Summary of de novo assembly of transcriptome sequence reads without reference genome**

**Samples**	**Total raw reads**	**Total clean reads**	**Total clean nucleotides (nt)**	**Q20 percentage**	**GC percentage**	**Type of gene detected**	**Total number**	**Total length (nt)**	**Mean length (nt)**	**N50**
RF1	48 335 970	44 166 280	4 416 628 000	96,59%	45,65%	Raw sequences	68 768	32 762 368	476	1284
						Consensus sequences	41 770	37 272 781	892	1627
RF2	49 872 928	45 562 458	4 556 245 800	96,55%	45,91%	Raw sequences	68 357	33 018 028	483	1289
						Consensus sequences	41 881	37 472 313	895	1611

First column shows the total raw reads, the next columns shows the clean reads and nucleotids. Q20 percentage column shows the proportion of nucleotides with quality value larger than 20. GC percentage is proportion of guanidine and cytosine nucleotides among total nucleotides. The total number column shows the total sequences obtained, that represents the all raw sequences and consensus sequences, respectively. Next columns show the total nucleotides length and their mean length. The last column shows the N50 value (defined as the length for which the collection of all sequences of that length or longer contains at least half of the sum of the lengths of all sequences).

The two independent samples, (RF1 and RF2) were collected and, after DNase treatment, RNA integrity was confirmed using a triple check, Nanodrop™, Experion™ Automated Electrophoresis System, and gel electrophoresis.

### *De novo* assembly of sequence reads without a reference genome

Reads were assembled using Trinity [26] and then, sequences were clustered using the TIGR Gene Indices clustering tools (TGICL). TGICL [28] was used *to join further sequences and remove any redundant sequences.*

So, the result of clustering was that from 68,768 and 68,357 raw sequence reads were generated; after clustering 41,770 and 41,881 total consensus sequences were generated respectively (Table 1).

Gene family clustering was performed such that the consensus sequences were divided into two classes. One class comprised clusters, for which the prefix CL followed by the cluster id and the number of contigs in each cluster was given (Additional file 1: Table S1). In any one cluster, there were several consensus sequences for which similarity between the consensus sequences was more than 70%. The other class comprised singletons, for which the prefix Unigene was given.

Altogether, considering both repetitions, 42,062 different consensus sequences were detected. Among them 21,903 were singletons, and 20,159 others were grouped into 7,610 different clusters.

The diagram in Additional file 2 shows the distribution of raw sequences and of the consensus sequence lengths ranging from 200 bp to more than 3,000 bp in both samples. The most abundant raw sequences were 200 bp (over 38,000) and the least abundant were 3000 bp (121); sequences over 3000 bp were grouped together. For the consensus sequences, the most abundant were 200 bp (over 7000), and the least abundant were 300 bp (150). The number of sequences decreased as the length increased (Additional file 2).

Consensus sequences were aligned with Blastdb using Blastx (evalue < 0.00001) [29]. Sequence orientations were determined according to the best hit in the database. The orientation and CDS of sequences that had no hit in blast were predicted using ESTScan [30].

### Annotation and classification of *Rubus* sp. consensus sequences

For annotation, the consensus sequences were first searched using BLASTX against the NCBI ‘non-redundant’ database (Nr) [31] using a cut-off E-value of 0.000001.

To search for the maximum number of similar genes, after using the Nr database, the NCBI’s NT database [31], Swiss Institute of Bioinformatics databases (Swiss-Prot) [32], Kyoto Encyclopedia of Genes and Genomes (KEGG) [33], Clusters of Orthologous Groups of proteins (COG) [34], Gene Ontology (GO) [35] databases were used. First, several databases were used to annotate each gene. In each database, two criteria were used, the score and the evalue. The evalue was set to discard alignments with statistical significance (NCBI minimum score = 58, evalue = 0.000001; and Swissprot minimum score = 30, evalue = 0.00001). Each gene was analyzed independently, and the annotation was made according to these criteria; these data are shown in Additional file 1. The KEGG PATHWAY database records networks of molecular interactions in cells, and variants of them, specific to particular organisms. Pathway-based analysis helped to understand further the biological functions of genes. Pathway information for all annotated sequences was obtained from KEGG pathway annotations.

COG is a database where orthologous gene products are classified. Every protein in COG is assumed to have evolved from an ancestral protein, and the whole database is built on genes encoding proteins from species with complete genome sequences as well as the evolutionary relationships between bacteria, algae and eukaryotes. All consensus sequences were aligned to the COG database to predict and classify possible functions. It was possible to get Gene Ontology (GO) functional annotation from the NR annotation. GO offers three ontologies: molecular function, cellular component and biological process. The basic unit of GO is the GO-term. Every GO-term belongs to a type of ontology. Based on the NR annotation, the Blast2GO program was used [36] to get the GO annotation of all consensus sequences. WEGO software [37] was then used for GO functional classification and to understand the distribution of gene functions of the species at a macro level.

For functional annotation, 33,040, 32,762, 21,932, 20,134, 13,676, 24,168 consensus sequences were annotated using the NR, NT, Swiss-Prot, KEGG, COG, GO databases, respectively; in total 34,552 annotated sequences were identified. For protein prediction analysis, the number of CDS that mapped to the protein database was 32,540.

Among the annotated sequences, the species with the highest number of best hits were wild strawberry (*Fragaria vesca subsp. vesca*) (73.56% matched genes) and peach (*Prunus persica*) (15.25% matches) (Table 2). These results are consistent since strawberry and peach are the species closest to *Rubus* sp. with sequenced genomes, all belonging to the family *Rosaceae*.

**Table 2:** **Summary of annotations of assembled Rubus sp. consensus sequences**

**Species**	**Number of genes**	**Percentage**
*Fragaria vesca subsp. vesca*	25 418	73,56%
*Prunus persica*	5 270	15,25%
*Vitis vinifera*	435	1,26%
*Ricimus communis*	183	0,53%
*Glycine max*	160	0,46%
*Populus balsamifera subsp. trichocarpa*	147	0,43%
*Medicago truncatula*	139	0,40%
*Lycopersicon esculentum*	107	0,31%
*Rosa rugosa*	101	0,29%
*Arabidopsis thaliana*	101	0,29%
Others	2491	7,21%

The first column shows the species with the highest number of similar genes in descending order, the second column indicates de number of these annotated genes, and the last column show the percentage of genes with respect to the total annotated genes sequences.

**Figure 1:**
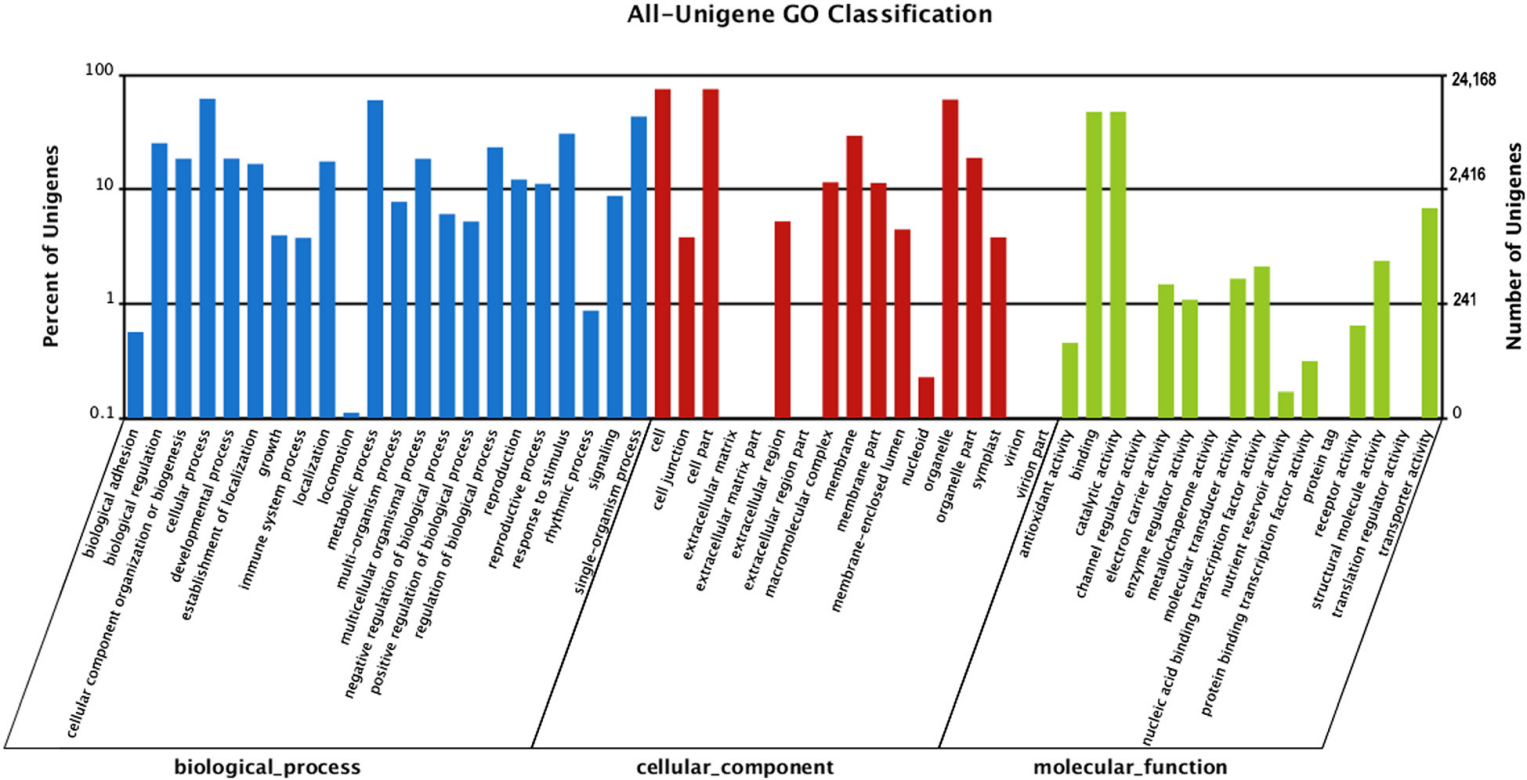
**Histogram of GO classifications of** ***Rubus*** **sp. consensus sequences ripe fruit.** Results are summarized for the three main GO categories: biological process, cellular component and molecular function. The left axis indicate the percent of sequences of each category, and right axis shows the total number of genes in each category.

Based on sequence homology, 24,168 *Rubus* sp. sequences were categorized into 40 functional groups, belonging to three main GO ontologies: molecular function, cellular component and biological process. Results showed a high proportion of genes from the categories of; “cellular process”, “metabolic process”, “cell” “organelle”, “catalytic” and “binding” with only a few genes related to “biological adhesion”, “immune system processes”, “growth”, “rhythmic process”, “nucleoid”, “antioxidant activity”, “nutrient reservoir activity”. No genes were clustered as “extracellular”, “virion”, “channel regulator activity”, “protein tag” or “translation regulator activity” (Figure 1).

To identify active biological pathways in ripe fruit of *Rubus* sp., the sequences were mapped to the reference canonical pathways in the Kyoto Encyclopedia of Genes and Genomes.

(KEGG). In total, 20,134 sequences were assigned to 128 KEGG pathways. The pathways with most representation were “metabolic pathways” (4,371 members), “Biosynthesis of secondary metabolites” (2,005 members), “plant-pathogen interaction” (1,471 members) and “RNA transport” (1,011 members) (Additional file 1). The 2,005 genes in the “Biosynthesis of secondary metabolites” category expressed in blackberry fruits will be useful for defining metabolic pathways for synthesis and turnover of compounds potentially beneficial to human health, and modifiable by plant breeding in Blackberry.

To further differentiate the NCBI nucleotide sequences and assembled sequences at the protein level, COG classification was undertaken to analyse the NCBI sequences.

The 13,676 assembled sequences were divided into 25 clusters according to NCBI COG classification (Additional file 3). The groups with the highest representation were found in the clusters R “general function prediction only”, K “transcription” and L “replication, recombination and repair” (Additional file 3).

To determine differential expression, once all reads were assembled and annotated, each gene expression level was normalized to its length for each replicate (C1 and C2). The gene expression level was calculated by using RPKM method [38] (Reads per kilobase transcriptome per million mapped reads), with the following formula: RPKM = 10^6^C/(NL/10^3^) which defines the expression of gene A , where C is the number of reads that are uniquely aligned to gene A , N is the total number of reads that are uniquely aligned to all genes, and L is the number of bases in gene A. The RPKM method is able to eliminate the influence of different gene lengths and sequencing discrepancies in the calculation of gene expression. Therefore, the calculated gene expression can be used directly for comparing the difference of gene expression among samples. Normalized data from C1 was plotted against data from C2; low dispersion in the plot indicated high repetitivity in expression between samples. Gene expression levels showed high similarity between biological replicates, RF1 and RF2 (Additional files 3 and 4). Most genes showed no significant differences between the samples, suggesting the results were reliable. Therefore, the calculated FPKM gene expression values can be directly compared between genes and, for any given gene, between samples.

Finally, SSRs were detected using MISA software [20], using the sequences as a reference (Additional file 5). Predominant SSRs were dinucleotides (over 4000), followed by trinucleotides (over 3000), mononucleotides (1200), hexanucleotides (365) and similar amounts of tetra and pentanucleotides (Additional file 5). Despite the importance of these sequences to predict variability in different organisms [39] no further analysis has been undertaken with these data in the present study, but the sequences are available in EBI databases (PRJEB6680), to use as markers for improvement of blackberry quality. Such SSRs will be useful as molecular markers for assaying the functional diversity in natural populations or germplasm collections, evolutionary studies and for breeding projects.

### *De novo* assembly of sequence reads using the reference genome from strawberry

Since the species distribution of NR annotation showed that 25,418 genes (77,5%) had the highest similarity with *Fragaria vesca* subspecies *vesca*, a reanalysis was carried out, aligning the blackberry reads to this reference genome to obtain a more accurate analysis of the ripe blackberry transcriptome.

Primary sequencing data produced by Illumina HiSeq TM 2000, (raw reads), was subjected to quality control (QC), to determine whether a resequencing step was needed. Raw reads were filtered into clean reads and aligned to the reference sequences with SOAPaligner/SOAP2 [27]. Then, the distribution of reads on reference genes and coverage analysis was done. The quality control was positive for both samples (Additional file 6), and therefore further analysis was undertaken.

The genome map rate and gene map rate were very low (lower than 7%) because, even though strawberry and blackberry belong to the family *Rosaceae*, they are quite distinct species and the alignment using the SOAP software was very strict (no more than 5 mismatches were allowed in the alignment) (Table 3). The alignment parameters were strict because we wanted to detect only the most similar genes, to compare this analysis with that undertaken with the first strategy. Although the number of genes was not as high as expected, (12,077 genes had high similarity to strawberry genes), a sufficient number were detected to allow comparative analyses. Ontology (GO) enrichment analysis and pathway enrichment analysis were undertaken, but, the results were not as representative nor complete as in the first analysis.

**Table 3:** **Summary of** ***de novo*** **assembly of transcriptome sequence reads with reference genome**

**Sample**	**Clean reads**	**Genome map rate**	**Gene map rate**	**Expressed gene**	**Alternative splicing**	**SNP**
RF1	44166280	4.42%	7.06%	11807	462	67521
RF2	45562458	4.31%	6.89%	12077	459	67845

Clean reads, Genome map rate (%), Gene map rate (%), number of expressed genes, the number of alternative splicing events and the number of Single-nucleotide polymorphism (SNP) are shown in each column for blackberry Ripe Fruit samples (RF1 and RF2).

**Figure 2:**
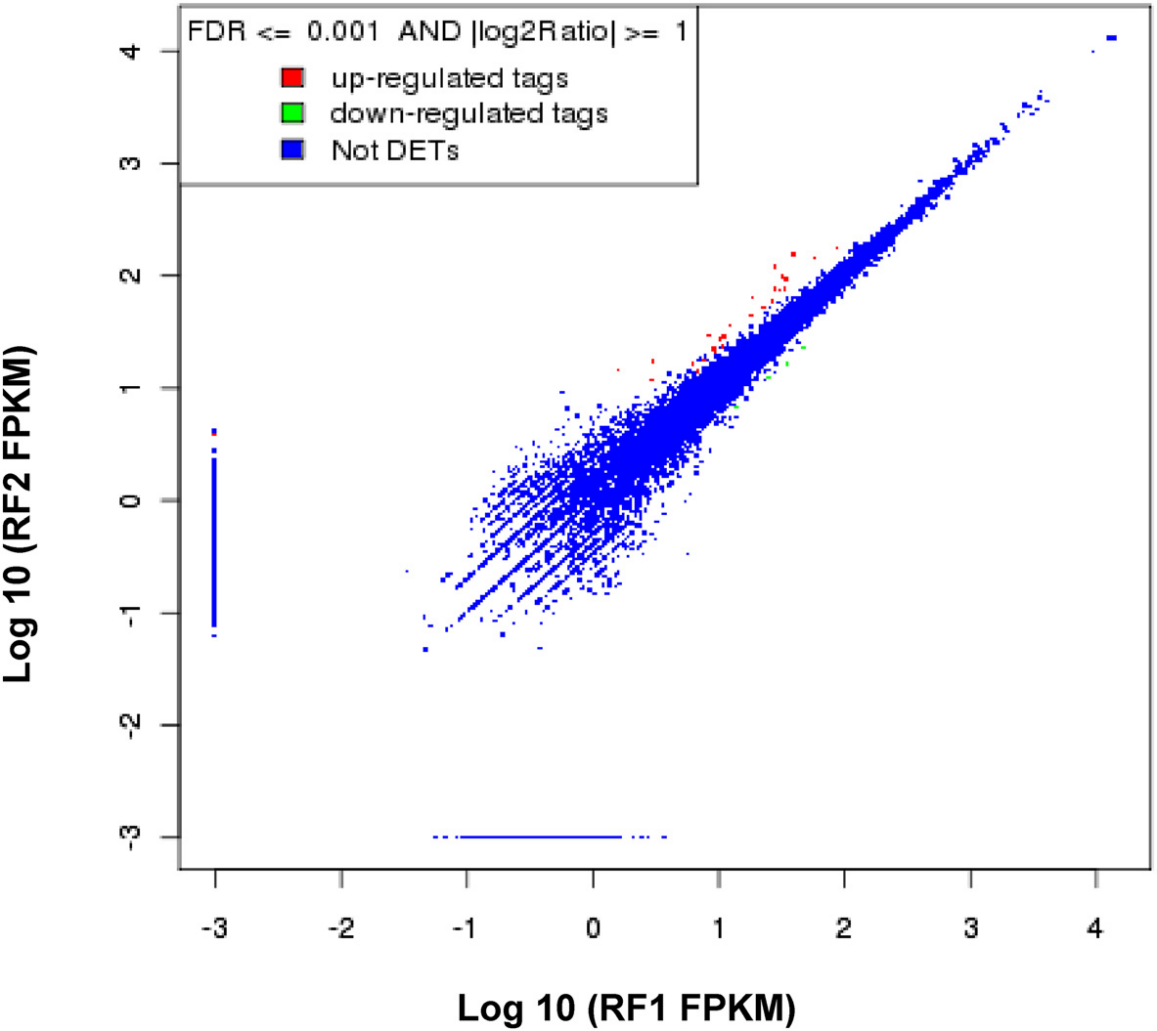
**Figure of distribution of differentially expressed genes.** X-axis (RF1) and Y-axis (RF2) shows the logarithm value of normalized expression of each gene in FPKM (Fragments per kb per Million fragments), for the two blackberry Ripe Fruit samples (RF1 and RF2) using *Fragaria* as reference genome. Red (Up) and green (down) dots indicate significantly different expression (FDR ≤ 0.001 and log2Ratio ≥ 1), and blue dots indicates no significant differences.

The expression levels of sequences were similar in both replicates RF1 and RF2 (Figure 2); only 31 genes had significantly different values (0.24%), suggesting highly reproducible results.

Single-nucleotide polymorphism (SNP) analysis was done with SOAPaligner/SOAP2 [27]. In samples RF1 and RF2, 67,521 SNPs and 67,845 SNPs were detected, respectively (Additional file 7).

### Comparison of strategies used to analyse the blackberry transcriptome *de novo*

Our initial analysis strategy (alignment using blastx with any plant sequence in the databases) produced a large number of annotated genes: 34,552 from a total of 42,062 assembled genes (82.14% of genes). This provides a significant database for berry breeders. All the classifications (COG, KEGG, etc.) provide new tools and resources for research on fruit development and bioactives. However, functional assignment of genes based on similarity to genes in other plant species should be undertaken with caution, especially if the comparator species are taxonomically distant from blackberry, such as *Populus balsamifera subsp. Trichocarpa*, *Medicago truncatula*, *Lycopersicon esculentum*, which all showed some genes with high similarity to those of blackberry (147, 139 and 107 genes respectively).

The second analysis (alignment with the closest sequenced genome *Fragaria vesca* subspecies *vesca*) resulted in a lower number of expressed genes, 12,077. Since very high stringency was set for this alignment with strawberry (on average less than 5 mismatches per gene), it is very likely that matched sequences have equivalent functions in the two species.

The combination of the two strategies for assembly and analysis of RNA-seq data adds value to the dataset for diverse applications.

### Study of putative chimeras

*Rubus* sp. Var Lochness is a tetraploid hybrid [40], and consequently there is a risk of chimeric contigs from assembling the NGS data. However, Trinity is reliable in assembling genes from different chromosomes and avoiding chimeras, especially when the hybrid has been derived from different species [26].

To test for putative chimeras, the CDS of one gene encoding *Chalcone Synthase* (*CHS*) was selected as a representative example for its role in biosynthesis of flavonols and anthocyanins, that are greatly accumulated in blackberries. The CDS was cloned from fresh tissue by designing primers (Additional file 8) for both ends of the known sequence; the CDS were cloned in pGEMT and several clones were sequenced.

All the sequences from the clones aligned with high scores (99%) with the two CHS contigs from the RNAseq data; however 33 nucleotides were different (2.9%) (Additional file 9) between the cloned sequences and the CHS contigs. These differences could be due to SNPs or to errors introduced during amplification by PCR or during the sequencing the genes. These sequence differences were clustered around 500 nucleotides from each end of the CDS.

Despite the high reliability of the software used to align the sequences to distinguish homologs of different chromosomes [41] and our results, that suggest that CHS is not chimeric, this represents a single test case, and a deeper analysis on more genes should be carried out, to rule out the occurrance of chimeric genes resulting from mistakes in alignment of transcripts in this tetraploid variety.

### Expression of the contigs estimated by qRT-PCR

RNA-seq analysis showed that more than 13,000 genes of the blackberry transcriptome are clustered in different contigs. This could be problematic for primer design for RT-qPCR analysis, since design of primers that amplify only one of the contigs encoding a specific protein, instead of all the copies of that gene, could give misleading expression data. To check if this is a real problem, three pairs of primers were designed for the CHS gene. The first two pairs were designed using the zone with high SNP frequency between the two contigs encoding CHS in blackberry (the first 500 bp, Additional file 9). Consequently, these primers should monitor the transcript levels of each contig encoding CHS but not the combined expression of both genes (Additional file 8). The third pair of primers was designed within the sequence conserved between the two genes; accordingly this third pair should report the total expression of the CHS genes.

**Figure 3:**
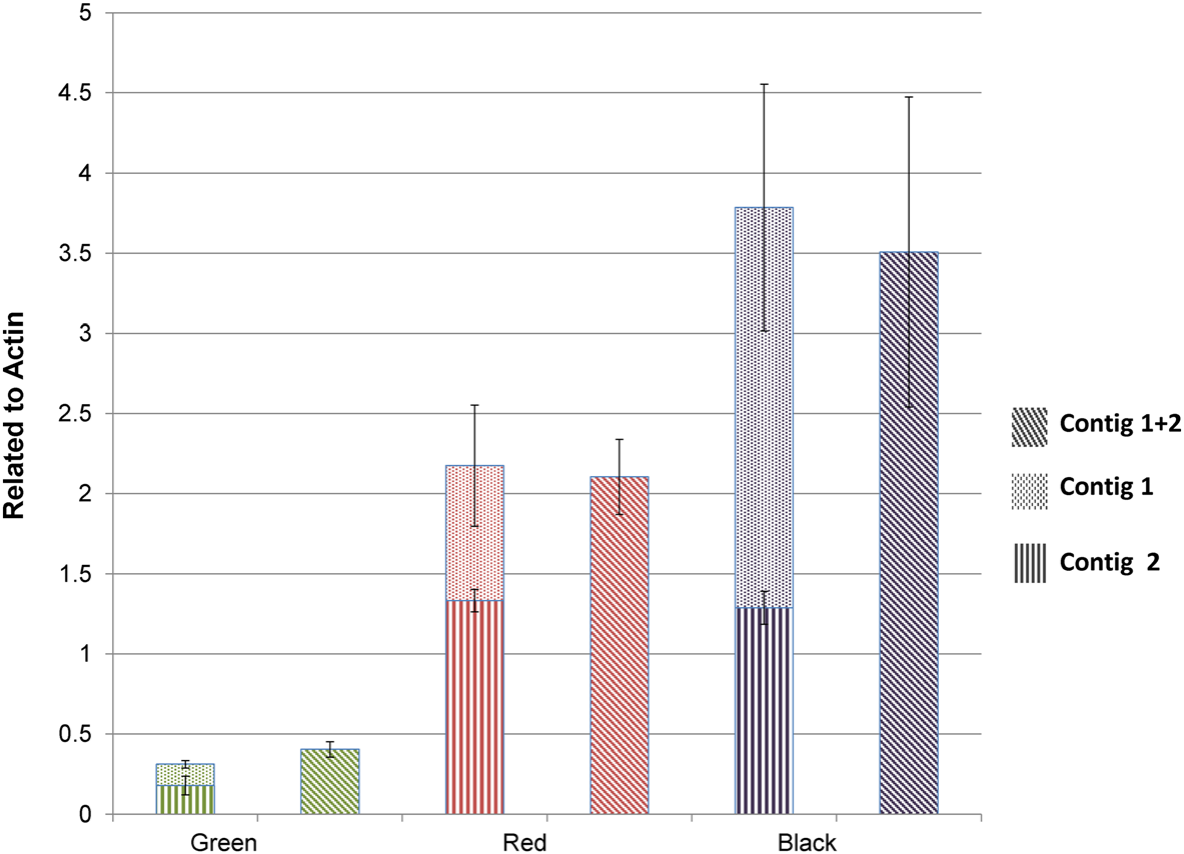
**Expression levels of** ***CHS*** **relative to actin for the two** ***CHS*** **contigs of blackberry, amplified independently and combined using different primer pairs.** Y-axe indicates the expression level compared to actin. Slant line background bars indicate the expression level using the primers of the common fraction of both contigs. Vertical line and dots background bars indicate the expression level using the primers of the differential fraction of both contigs.

RT-qPCR showed that the expression reported by this third primer pair (Contig1 + 2) was equal to the sum of the RT-qPCR products of the two primer pairs which amplified Contig1 and Contig2 separately, during three stages of ripening of blackberry fruit (green, red and black) (Figure 3). These data illustrate how gene expression analysis is best undertaken for tetraploid varieties such as blackberry var LochNess.

Although these studies represent assays of a single gene for chimeras, the degree of polymorphism between the two CHS contigs was such (10 mismatches per contig of 1200 nt) that data on this gene likely represent the top end of the problem, where single nucleotide differences would impact the proteins encoded, since on average 5SNPs were found per contig. Consequently chimeras existing in other contig pairs are less likely to impact the sequence of the encoded protein than for CHS.

## Conclusions

The blackberry transcriptome data provide a resource that will enhance comparative studies between different berry crops which are of increasing economic and social importance due to their healthy properties. Consequently these transcriptome data and analyses provide an important new resource for biological research. The genes we have identified provide candidates for resistance genes against RNA viruses, fungal or bacterial pathogens as well as many genes encoding enzymes of flavonoid metabolism which are key to the health-promoting effects of many berries. The SSRs and SNPs identified here will constitute an important resource for mapping and marker-assisted breeding of ripe fruit quality traits in *Rubus* sp. and closely related crop species.

Two different strategies have been used to analyse sequence, assemble and annotate a new transcriptome *de novo* without a reference genome or transcriptome. We have demonstrated that the techniques are both robust and complementary and should be used in accordance with the research objectives. We show that the transcriptome of a non-model polyploid species, in this case, a tetraploid, can be sequenced, assembled and annotated avoiding high levels of artificial chimeras. The analysis of expression of several contigs of the same gene can inform expression analyses of both individual genes and all the genes encoding proteins of particular interest.

## Methods

### Plant materials and RNA isolation

The *Rubus sp.* var. Lochness plants used in this study were planted at production fields of the company Agricola El Bosque (Lucena del Puerto, Huelva, Spain). Plants and greenhouses were kindly provided by the company and all were handled according to regular agricultural practices [42]. Plants were grown under “winter cycle” that is, after an artificial cold period, from July to November 2013 under natural light conditions.

Prior to RNA extraction, samples were removed from the −80°C freezer and ground to a fine powder with liquid nitrogen using a sterilized mortar and pestle Total RNA was isolated from red fruits with PureLink™ Micro-to-Midi Total RNA Purification System (Invitrogen™), and after DNase treatment and confirmation of RNA integrity using a triple check, Nanodrop™, Experion™ Automated Electrophoresis System, and gel electrophoresis, the total RNA was used in mRNA preparation, fragmentation and cDNA synthesis.

Beads with oligo(dT) were used to isolate poly(A) mRNA from total RNA (Qiagen GmbH, Hilden, Germany).

### Synthesis of cDNA and sequencing

RNA-seq library preparation and sequencing was carried out by Beijing Genomics Institute BGI (Hong-Kong, China). Following purification, the mRNA was fragmented using divalent cations at elevated temperature. Taking these short fragments as templates, the first-strand cDNA was synthesized using random hexamer primers and Superscript TM III (Invitrogen™, Carlsbad, CA, USA). The second strand cDNA was synthesized using buffer, dNTPs, RNaseH and DNA polymerase I. Short fragments were purified with a QiaQuick PCR extraction kit (Qiagen) and resolved with EB buffer for end reparation and poly(A) addition. The short fragments were then connected using sequencing adapters.

After agarose gel electrophoresis, suitable fragments were used as templates for PCR amplification. During the QC steps, an Agilent 2100 Bioanaylzer and an ABI StepOnePlus Real-Time PCR System were used in quantification and qualification of the sample library. Finally, the library (200 bp insert) was sequenced using Illumina HiSeqTM 2000 (Illumina Inc., San Diego, CA, USA). The paired-end library was prepared following the protocol of the Illumina TruSeq RNA Sample Preparation Kit (Illumina). The library was linked to a paired-end flow cell containing complementary adapters, and then bound fragments were amplified to create overlapping “clusters”. The adapters were designed to allow selective cleavage of the forward DNA strand after resynthesis of the reverse strand during sequencing. The copied reverse strand was then used to sequence from the opposite end of the fragment. The raw reads were cleaned by removing adaptor sequences, empty reads and low quality sequences.

### Transcriptome de novo assembly

Two different strategies were followed to assemble the transcriptome; 1) *de novo* assembly using Trinity [26] and a later annotation with Blast using different databases, 2) aligning the raw data to the reference sequence with SOAPaligner/SOAP2 [27], using as the reference the sequence of wild strawberry (*Fragaria vesca* subesp. *vesca*) a species of same family (*Rosaceae*), but from a different genus, *Fragaria*.

In undertaking the first analysis, there was no reference genome for the alignment of the sequenced reads. The RNA-Seq reads were assembled into transcripts using Trinity [26], a reference genome-independent assembler [43]. The transcript identification by Trinity is divided into three steps: Inchworm, Chrysalis and Butterfly. Together, they assemble the RNA-seq reads into sequences, cluster the sequences, construct de Bruijn graphs [44] for each cluster (representing the transcriptional complexity for a gene), partition the reads among each graph, and finally trace the paths in each graph to report full-length transcripts, for alternatively spliced isoforms as well as for paralogous genes. Trinity has been found to efficiently reconstruct the transcriptome, inclusive of the splicing events and transcripts resulting from recent duplication events, better than other available *de novo* transcriptome assemblers [26].

For the second strategy, the strawberry genome and gene information were downloaded from the Washington State University and Clemson University website (http://www.rosaceae.org), funded by the 2009 USDA NIFA Specialty Crop Research Initiative Program. Sequencing-received raw image data were transformed by base culling into sequence data. *De novo* transcriptome assembly was aligned to the wild strawberry genome (*Fragaria vesca* subesp*. vesca*) using the assembling program SOAPaligner/SOAP2 [27]; all genes with more than five mismatches were discarded from analysis.

### Annotation and classification of consensus sequences

Sequences were used for BLAST searches and annotation against an NCBI Nr protein database (NCBI non-redundant sequence database) using an E-value cut-off of 0.00001 (E-value ≤ 0.00001). Consensus sequences were further aligned by BLASTX to protein databases such as Swiss-Prot, KEGG and COG, retrieving proteins with the highest sequence similarity with the given sequences along with the functional annotations for their proteins. If results of different databases conflicted, a priority order of Nr, Swiss-Prot, KEGG and COG was followed.

The coding region sequences were then determined for proteins with the highest ranks using BLAST. The Blast2GO program was used to obtain GO annotations for the sequences, as well as for KEGG and COG analysis [36]. The WEGO software was then used to perform GO functional classification of all sequences to view the distribution of gene functions of the species at the macro level [37]. The analysis mapped all of the annotated sequences to GO terms in the database and calculated the number of sequences associated with every term.

The gene expression level was calculated by using RPKM method [38] (Reads per kilobase transcriptome per million mapped reads), and the formula is as follows: RPKM = 106C/(NL/103). Given to be the expression of gene A, C to be number of reads that are uniquely aligned to gene A , N to be total number of reads that are uniquely aligned to all genes, and L to be number of bases on gene.

### Study of putative chimeras

The Chalcone synthase gene that was sequenced by RNA-seq was isolated from blackberry fruit tissue using the primers shown in Additional file 8. Primer design used the online software Primer 3 Plus [45]. First strand cDNA was synthesised using SuperScript™ III (Invitrogen) with 1–2 μg of total RNA accoding to the manufacturer’s instructions. The cDNA product was diluted to 10 ng/μg based on the initial amount of RNA.

PCR reactions were undertaken using G-Storm Thermal Cycler (Kapa Biosystems). The reaction mixture normally consisted of 10–20 ng of DNA template, 0.1 μM each of the forward and reverse primer, 100 μM of dNTPs, Taq DNA polymerase (1 unit) and Taq buffer in a total volume of 15 μL. The standard PCR protocol was: initial denaturation (4 min at 94°C), followed by 25–35 cycles of denaturation (45 seconds at 94°C), annealing (30 seconds at 60°C) and extension (60 seconds at 72°C), and final extension( 5 minutes at 72°C). For cloning PCR procedure, the protocol was similar to that described above except a tiny amount of bacterial colony was used to replace the template DNA. To purify DNA from PCR reactions, a QIAquick PCR Purification Kit (Qiagen) was used.

Heat shock transformation was used to transform E.coli (DH5α) cells with desired plasmids. Plasmid DNA was isolated from 3–5 mL of culture grown overnight under the appropriate antibiotic selection. Plasmid DNA isolation was done using QIAprep® Miniprep Kit (Qiagen). The concentration of DNA and RNA were quantified by using a NanoDrop 2000C UV–vis Spectrophotometer (Thermo). Once the plasmid was isolated, it was sequenced by Eurofins Genomics (UK).

### RT-qPCR of gene expression during the three stages of blackberry fruit ripening

RT-qPCR was performed using SYBR® Green JumpStart™ Taq ReadyMix™ (Sigma). All RT-qPCRs were performed using an Opticon 2 Real Time PCR machine (MJ Research): 10 min at 95°C and then 40 cycles consisting of 20 sec at 95°C, 20 sec at 60°C and 20 sec at 72°C, followed by 10 min at 72°C. A gene encoding actin was used as a reference.

To evaluate RT-qPCR values, oneway analysis of variance was performed. When differences were significant, the least significant differences (LSD) post hoc test was also performed [46] using the software Statgraphics plus 5.1 for Windows.

### Availability of supporting data

All data sets are available in the EBI database (accession number: PRJEB6680).

## Additional files

Additional file 1: Table S1. Summary of *all sequences* assembled and annotated by NR (A) by NT, Swiss-Prot and COG (B) and by KEGG and GO (C). Parameters recorded: Gene length, Raw fragment expression and FPKM (Fragments per kb per Million fragments) for (RF1 (Ripe fruit sample 1) and RF2 (Ripe fruit sample 2). And the score, e-value, and ID for each database respectively: NR (Table S1.A) by NT, Swiss-Prot and COG (Table S1.B) and by KEGG and GO (Table S1.C). Additional file 2:
**Statistical analysis of de novo assembly of** ***Rubus*** **sp. sequences.** The length distributions of raw sequences (A, B) and consensus sequences (C,D) are shown, for RF1 (A,C) and RF2 (B,D) are described of Ripe fruit of blackberry (*Rubus* sp. Var. Lochness), above each column is indicated the number of genes of each length range. Additional file 3:
**Histogram of COG classifications of assembled of** ***Rubus*** **sp. Ripe fruit.** Results are presented for the 25 main COG categories. The left axis indicate number of genes in each category. Additional file 4:
**Figure of distribution of differentially expressed genes.** X-axis (RF1) and Y-axis (RF2) shows the logarithm value of normalized expression of each gene in FPKM (Fragments per kb per Million fragments), for the two ripe blackberry fruit samples (RF1 and RF2) without reference genome. Red (Up) and green (down) dots indicate significantly different expression (FDR≤0.001 and log2Ratio≥1), and blue dots indicates no significant differences. Additional file 5:
**Histogram of SSR (Simple Sequence Repeats) motif of assembled ripe fruit** ***Rubus*** **sp.** Y-axis indicate the number of SSR motif in each category that is indicated in X-axis. Above each column is shown the number of genes of each category. Additional file 6:
**Quality distribution of bases along reads for the two blackberry Ripe fruit samples (RF1 and RF2).** Horizontal axes show positions along reads. Vertical axes show quality values. Each dot in the image represents the quality value of the corresponding position along reads. If the percentage of the bases with low quality (<20) is low, then the sequencing quality of this lane is good. Additional file 7:
**Statistical analysis of Single-nucleotide polymorphism (SNP) types in for the two blackberry Ripe Fruit samples (RF1) (B) and RF2 (B).** The ‘dark grey’ bars show transitions (A/G;C/T) and ‘grey’ bars show transversions (A/C;A/T;/G;G/T). Y-axis indicate the number of SSR motif in each category. Additional file 8:
**Primers used to isolate the CHS gene and RT-qPCR.**
Additional file 9:
**Position of Single-nucleotide polymorphism (SNPs) in similar Chalcone Synthase (** ***CHS*** **)** **contig sequences.** First column shows the position of the SNPs in the contig, the second column indicates the SNP in the sequence obtained by RNAseq and the remaining columns show the SNP in the sequences obtained by cloning PCR product.

## References

[1] Kalkman C (2004). Rosaceae. Flowering plants · dicotyledons.

[2] Martin C, Zhang Y, Tonelli C, Petroni K (2013). Plants, diet, and health. Annu Rev Plant Biol.

[3] Kaume L, Howard LR, Devareddy L (2012). The blackberry fruit: a review on its composition and chemistry, metabolism and bioavailability, and health benefits. J Agric Food Chem.

[4] Velasco R, Zharkikh A, Affourtit J, Dhingra A, Cestaro A, Kalyanaraman A, Fontana P, Bhatnagar SK, Troggio M, Pruss D (2010). The genome of the domesticated apple (*Malus domestica* Borkh.). Nat Genet.

[5] Shulaev V, Sargent DJ, Crowhurst RN, Mockler TC, Folkerts O, Delcher AL, Jaiswal P, Mockaitis K, Liston A, Mane SP (2010). The genome of woodland strawberry (*Fragaria vesca*). Nat Genet.

[6] Verde I, Abbott AG, Scalabrin S, Jung S, Shu S, Marroni F, Zhebentyayeva T, Dettori MT, Grimwood J, Cattonaro F (2013). The high-quality draft genome of peach (*Prunus persica*) identifies unique patterns of genetic diversity, domestication and genome evolution. Nat Genet.

[7] Pindo M, Montanari S, Cestaro A, Velsaco R (2012). A draft genome sequence of European pear (*Pyrus communis* L.‘Bartlett’). 6th Rosaceous Genomics Conference (RGC6), Mezzocorona (TN), 30th September-4th October 2012: 2012.

[8] Genome Database for Rosaceae (GDR). [http://www.rosaceaeorg].

[9] Focke WO (1910). Species ruborum.

[10] Monasterio-Huelin E (1992). Revisión taxonómica del género *Rubus* L (*Rosaceae*) en la Península Ibérica e Islas Baleares.

[11] Alice LA, Campbell CS (1999). Phylogeny of *Rubus* (*rosaceae*) based on nuclear ribosomal DNA internal transcribed spacer region sequences. Am J Bot.

[12] Brooks RM (1997). The brooks and olmo register of fruit & nut varieties.

[13] Kassim A, Poette J, Paterson A, Zait D, McCallum S, Woodhead M, Smith K, Hackett C, Graham J (2009). Environmental and seasonal influences on red raspberry anthocyanin antioxidant contents and identification of quantitative traits loci (QTL). Mol Nutr Food Res.

[14] Zheng D, Schröder G, Schröder J, Hrazdina G (2001). Molecular and biochemical characterization of three aromatic polyketide synthase genes from *Rubus idaeus*. Plant Mol Biol.

[15] Zheng D, Hrazdina G (2008). Molecular and biochemical characterization of benzalacetone synthase and chalcone synthase genes and their proteins from raspberry (*Rubus idaeus* L.). Arch Biochem Biophys.

[16] Graham J, Smith K, MacKenzie K, Jorgenson L, Hackett C, Powell W (2004). The construction of a genetic linkage map of red raspberry (*Rubus idaeus* subsp. idaeus) based on AFLPs, genomic-SSR and EST-SSR markers. Theor Appl Genet.

[17] Zheng D, Hrazdina G (2010). Cloning and characterization of an expansin gene, RiEXP1, and a 1-aminocyclopropane-1-carboxylic acid synthase gene, RiACS1 in ripening fruit of raspberry (Rubus idaeus L.). Plant Sci.

[18] Kumar A, Ellis BE (2003). A family of polyketide synthase genes expressed in ripening *Rubus* fruits. Phytochemistry.

[19] Woodhead M, Weir A, Smith K, McCallum S, MacKenzie K, Graham J (2010). Functional markers for red raspberry. J Am Soc Hortic Sci.

[20] Varshney RK, Graner A, Sorrells ME (2005). Genic microsatellite markers in plants: features and applications. Trends Biotechnol.

[21] Simon SA, Zhai J, Nandety RS, McCormick KP, Zeng J, Mejia D, Meyers BC (2009). Short-read sequencing technologies for transcriptional analyses. Annu Rev Plant Biol.

[22] Adams MD, Kelley JM, Gocayne JD, Dubnick M, Polymeropoulos MH, Xiao H, Merril CR, Wu A, Olde B, Moreno RF (1991). Complementary DNA sequencing: expressed sequence tags and human genome project. Science.

[23] Lister R, Gregory BD, Ecker JR (2009). Next is now: new technologies for sequencing of genomes, transcriptomes, and beyond. Curr Op Plant Biol.

[24] Wang Z, Gerstein M, Snyder M (2009). RNA-Seq: a revolutionary tool for transcriptomics. Nat Rev Genet.

[25] Trapnell C, Williams BA, Pertea G, Mortazavi A, Kwan G, van Baren MJ, Salzberg SL, Wold BJ, Pachter L (2010). Transcript assembly and quantification by RNA-Seq reveals unannotated transcripts and isoform switching during cell differentiation. Nature Biotechnol.

[26] Grabherr MG, Haas BJ, Yassour M, Levin JZ, Thompson DA, Amit I, Adiconis X, Fan L, Raychowdhury R, Zeng Q (2011). Full-length transcriptome assembly from RNA-Seq data without a reference genome. Nature Biotechnol.

[27] Li R, Li Y, Kristiansen K, Wang J (2008). SOAP: short oligonucleotide alignment program. Bioinformatics.

[28] Pertea G, Huang X, Liang F, Antonescu V, Sultana R, Karamycheva S, Lee Y, White J, Cheung F, Parvizi B (2003). TIGR Gene Indices clustering tools (TGICL): a software system for fast clustering of large EST datasets. Bioinformatics.

[29] Altschul SF, Madden TL, Schäffer AA, Zhang J, Zhang Z, Miller W, Lipman DJ (1997). Gapped BLAST and PSI-BLAST: a new generation of protein database search programs. Nucleic Acids Res.

[30] Iseli C, Jongeneel CV, Bucher P (1999). ESTScan: a program for detecting, evaluating, and reconstructing potential coding regions in EST sequences. ISMB.

[31] Pruitt KD, Tatusova T, Maglott DR (2007). NCBI reference sequences (RefSeq): a curated non-redundant sequence database of genomes, transcripts and proteins. Nucleic Acids Res.

[32] Bairoch A, Boeckmann B (1991). The SWISS-PROT protein sequence data bank. Nucleic Acids Res.

[33] Kanehisa M, Goto S (2000). KEGG: kyoto encyclopedia of genes and genomes. Nucleic Acids Res.

[34] Tatusov RL, Fedorova ND, Jackson JD, Jacobs AR, Kiryutin B, Koonin EV, Krylov DM, Mazumder R, Mekhedov SL, Nikolskaya AN (2003). The COG database: an updated version includes eukaryotes. BMC Bioinformatics.

[35] Consortium GO (2004). The Gene Ontology (GO) database and informatics resource. Nucleic Acids Res.

[36] Conesa A, Götz S, García-Gómez JM, Terol J, Talón M, Robles M (2005). Blast2GO: a universal tool for annotation, visualization and analysis in functional genomics research. Bioinformatics.

[37] Ye J, Fang L, Zheng H, Zhang Y, Chen J, Zhang Z, Wang J, Li S, Li R, Bolund L (2006). WEGO: a web tool for plotting GO annotations. Nucleic Acids Res.

[38] Mortazavi A, Williams BA, McCue K, Schaeffer L, Wold B (2008). Mapping and quantifying mammalian transcriptomes by RNA-Seq. Nat Methods.

[39] Legendre M, Pochet N, Pak T, Verstrepen KJ (2007). Sequence-based estimation of minisatellite and microsatellite repeat variability. Genome Res.

[40] Castillo NR, Reed BM, Graham J, Fernández-Fernández F, Bassil NV (2010). Microsatellite markers for raspberry and blackberry. J Am Soc Hortic Sci.

[41] Nakasugi K, Crowhurst R, Bally J, Waterhouse P (2014). Combining transcriptome assemblies from multiple de novo assemblers in the allo-tetraploid plant nicotiana benthamiana. PLoS One.

[42] Ramos-Solano B, Garcia-Villaraco A, Gutierrez-Mañero F, Lucas J, Bonilla A, Garcia-Seco D (2014). Annual changes in bioactive contents and production in field-grown blackberry after inoculation with *Pseudomonas fluorescens*. Plant Physiol Bioch.

[43] Martin JA, Wang Z (2011). Next-generation transcriptome assembly. Nat Rev Genet.

[44] de Bruijn NG, Erdos P (1946). A combinatorial problem. K Ned Akad Van Wet-P.

[45] Untergasser A, Nijveen H, Rao X, Bisseling T, Geurts R, Leunissen JA (2007). Primer3Plus, an enhanced web interface to Primer3. Nucleic Acids Res.

[46] Sokal RR, Rohlf F (1981). Biometry.

