# Supplementary figures and images for: RNA-Seq analysis and transcriptome assembly for blackberry (Rubus sp. Var. Lochness) fruit
Source: BMC Genomics. 2015 Jan 22;16(1):5. doi: 10.1186/s12864-014-1198-1 (PMC4311454; doi:10.1186/s12864-014-1198-1)

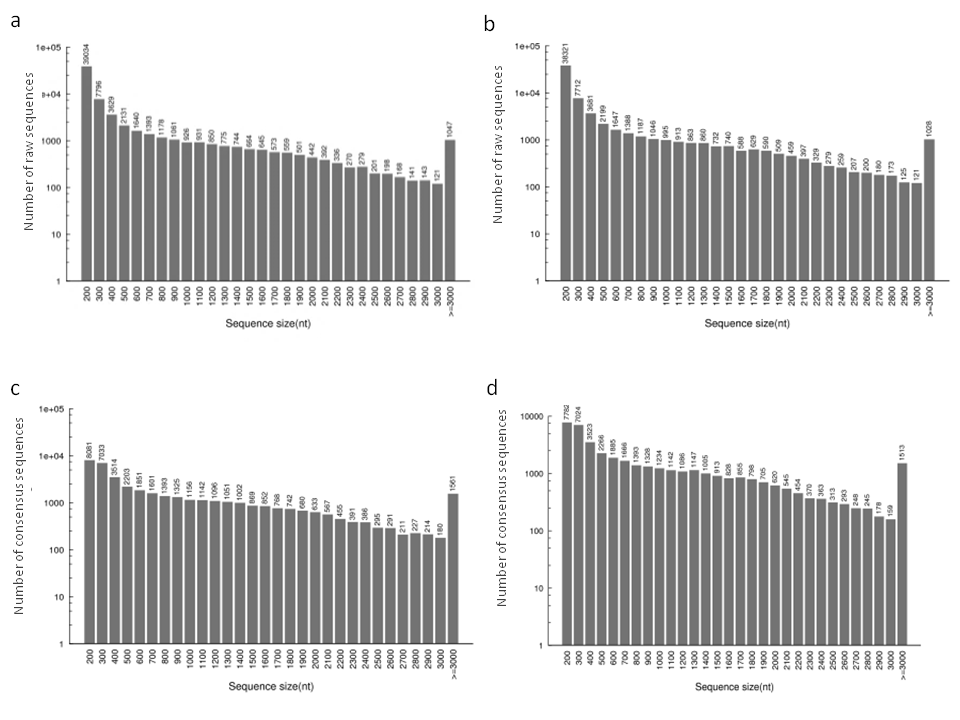

Supplement: Additional file 2: — Statistical analysis of de novo assembly of Rubus sp. sequences. The length distributions of raw sequences (A, B) and consensus sequences (C,D) are shown, for RF1 (A,C) and RF2 (B,D) are described of Ripe fruit of blackberry (Rubus sp. Var. Lochness), above each column is indicated the number of genes of each length range. [file 12864_2014_1198_MOESM2_ESM.tiff]

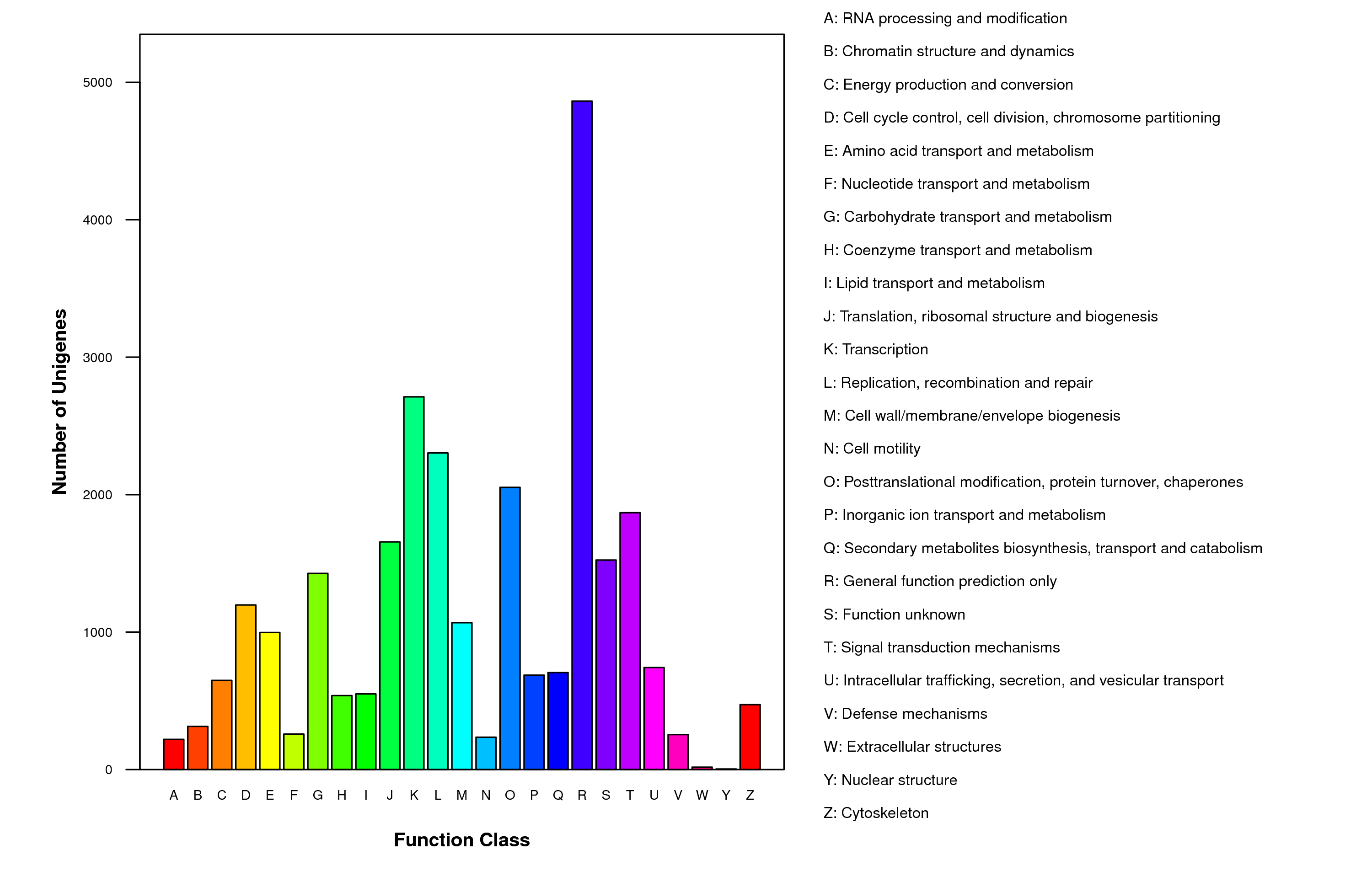

Supplement: Additional file 3: — Histogram of COG classifications of assembled of Rubus sp. Ripe fruit. Results are presented for the 25 main COG categories. The left axis indicate number of genes in each category. [file 12864_2014_1198_MOESM3_ESM.png]

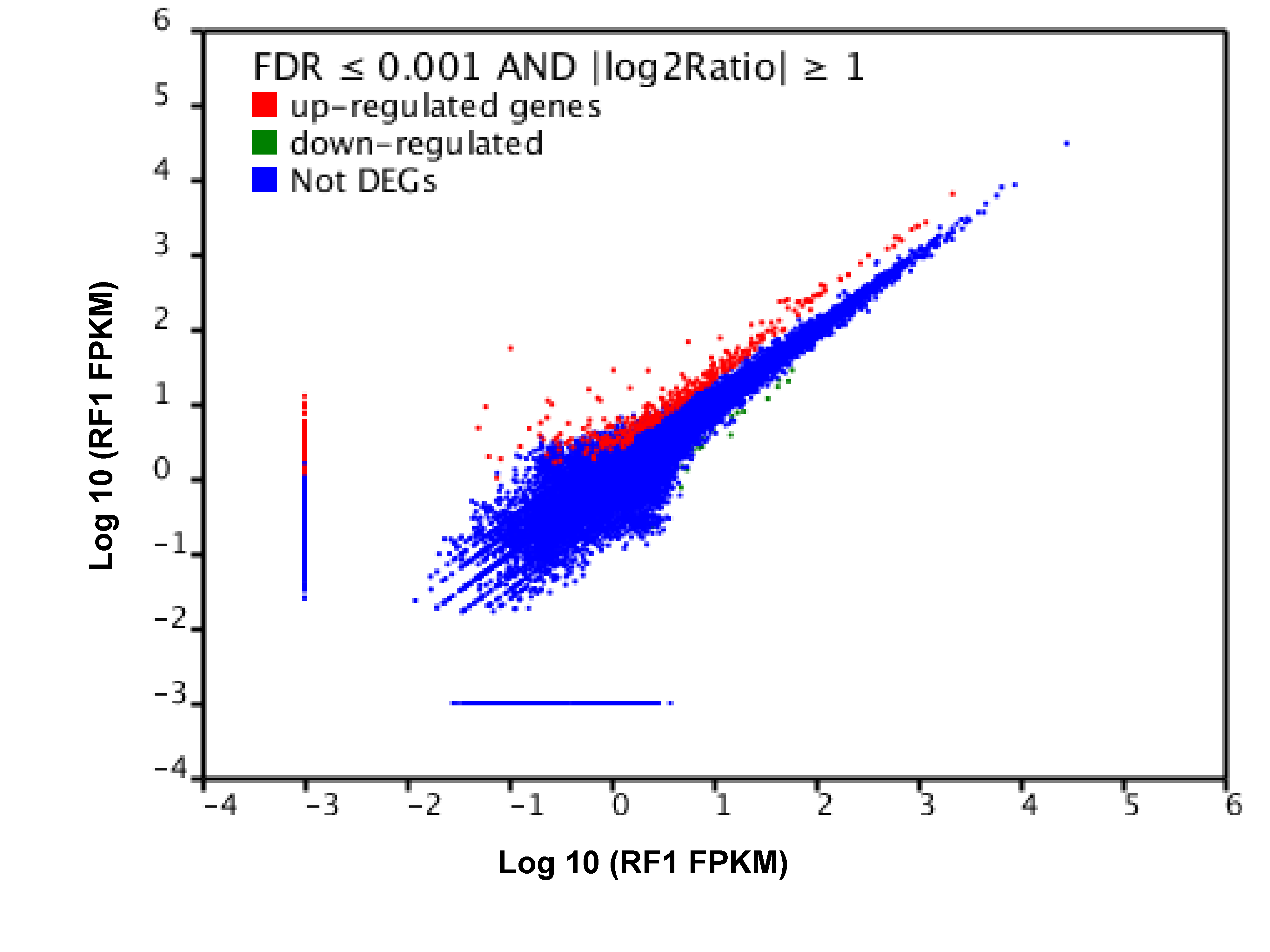

Supplement: Additional file 4: — Figure of distribution of differentially expressed genes. X-axis (RF1) and Y-axis (RF2) shows the logarithm value of normalized expression of each gene in FPKM (Fragments per kb per Million fragments), for the two ripe blackberry fruit samples (RF1 and RF2) without reference genome. Red (Up) and green (down) dots indicate significantly different expression (FDR≤0.001 and log2Ratio≥1), and blue dots indicates no significant differences. [file 12864_2014_1198_MOESM4_ESM.tiff]

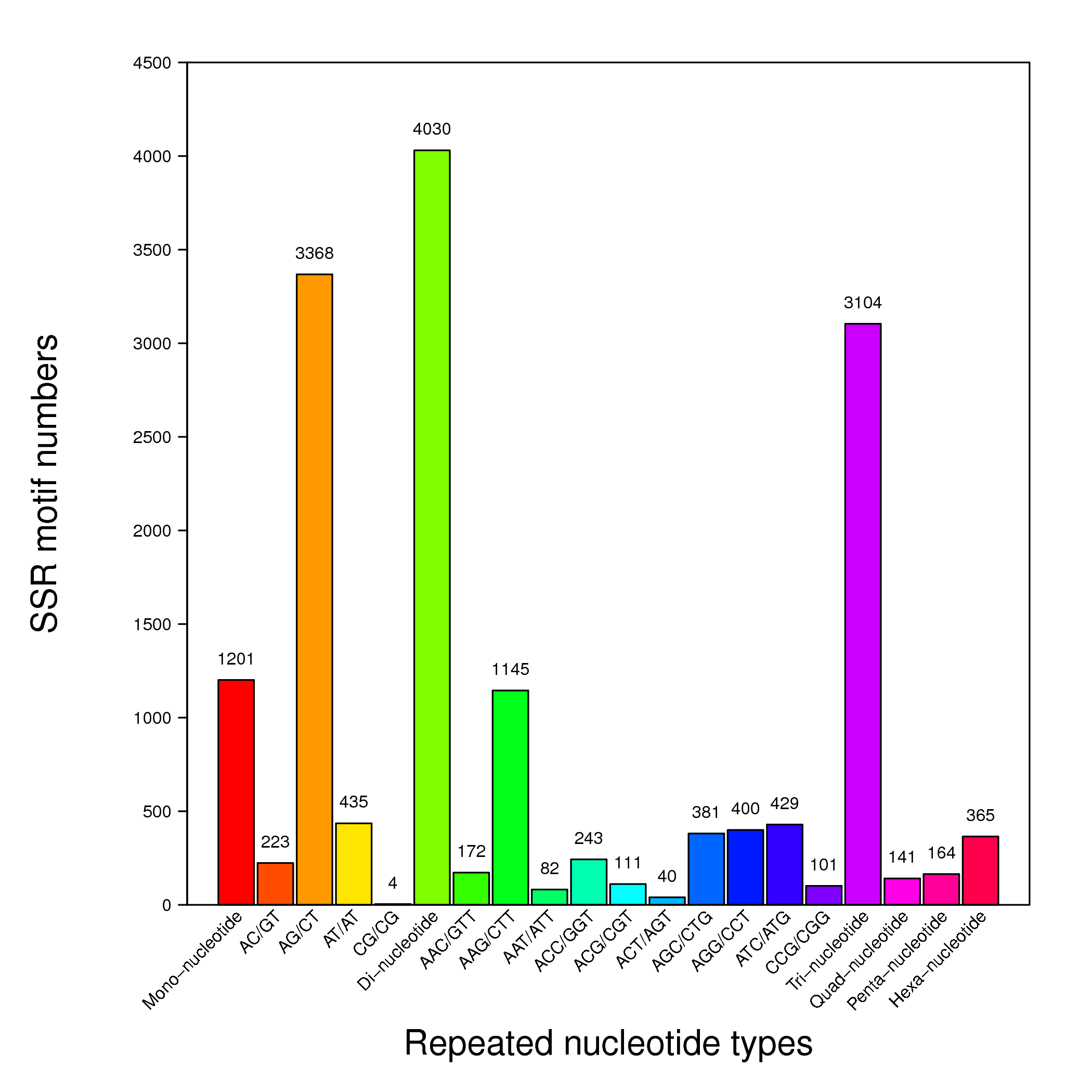

Supplement: Additional file 5: — Histogram of SSR (Simple Sequence Repeats) motif of assembled ripe fruit Rubus sp. Y-axis indicate the number of SSR motif in each category that is indicated in X-axis. Above each column is shown the number of genes of each category. [file 12864_2014_1198_MOESM5_ESM.png]

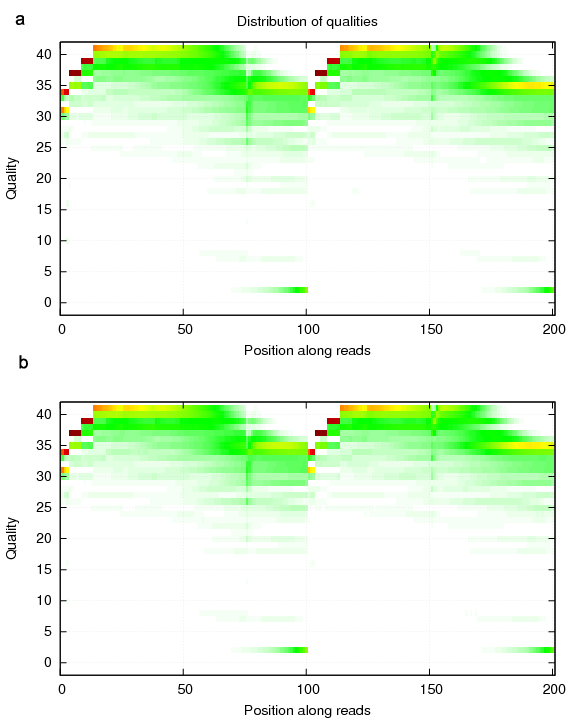

Supplement: Additional file 6: — Quality distribution of bases along reads for the two blackberry Ripe fruit samples (RF1 and RF2). Horizontal axes show positions along reads. Vertical axes show quality values. Each dot in the image represents the quality value of the corresponding position along reads. If the percentage of the bases with low quality (<20) is low, then the sequencing quality of this lane is good. [file 12864_2014_1198_MOESM6_ESM.tiff]

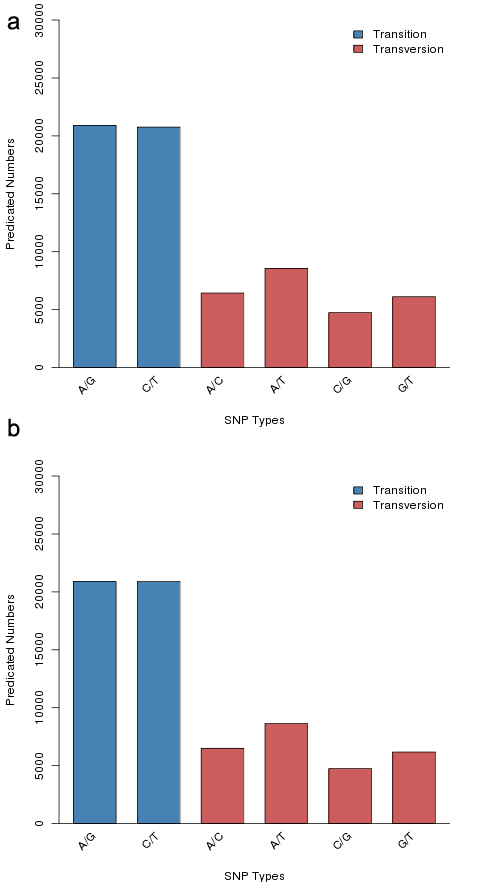

Supplement: Additional file 7: — Statistical analysis of Single-nucleotide polymorphism (SNP) types in for the two blackberry Ripe Fruit samples (RF1) (B) and RF2 (B). The ‘dark grey’ bars show transitions (A/G;C/T) and ‘grey’ bars show transversions (A/C;A/T;/G;G/T). Y-axis indicate the number of SSR motif in each category. [file 12864_2014_1198_MOESM7_ESM.tiff]
