# Supplementary material for: RNA-Seq analysis and transcriptome assembly for blackberry (Rubus sp. Var. Lochness) fruit
Source: BMC Genomics. 2015 Jan 22;16(1):5. doi: 10.1186/s12864-014-1198-1 (PMC4311454; doi:10.1186/s12864-014-1198-1)
Supplement: Additional file 8: — Primers used to isolate the CHS gene and RT-qPCR. [file 12864_2014_1198_MOESM8_ESM.pdf]

Primer	Sequence
pRuCHS-seq-F	ATGGTGACCGTCGAGGAAGTTCGC
pRuCHS-seq-R	AGTTGAAGCTGCCACACTGTGAAG
pRuContig1-qRT-F	GGCAACTCCACCCAACTGTATCT
pRuContig1-qRT-R	CGCTGGAATTTCTCTTTTAGCTC
pRuContig2-qRT-F	ACAGCAACTCCTCCCAACTGTGTT
pRuContig2-qRT-R	GGAATTTCTCCTTGAGCTCAGTC
pRuContig1+2-qRT-F	AGAAATTCCAGCGCATGTGT
pRuContig1+2-qRT-R	GTGCCATGTACTCGCACATACTA
