# Supplementary material for: RNA-Seq analysis and transcriptome assembly for blackberry (Rubus sp. Var. Lochness) fruit
Source: BMC Genomics. 2015 Jan 22;16(1):5. doi: 10.1186/s12864-014-1198-1 (PMC4311454; doi:10.1186/s12864-014-1198-1)
Supplement: Additional file 9: — Position of Single-nucleotide polymorphism (SNPs) in similar Chalcone Synthase ( CHS ) contig sequences. First column shows the position of the SNPs in the contig, the second column indicates the SNP in the sequence obtained by RNAseq and the remaining columns show the SNP in the sequences obtained by cloning PCR product. [file 12864_2014_1198_MOESM9_ESM.pdf]

Position of SNP	CHS RNAseq contig 1	CHS Cloned Sequence 1	CHS Cloned Sequence 2	CHS RNAseq contig 2	CHS Cloned Sequence 3	CHS Cloned Sequence 4
33	A	A	A	G	G	G
39	T	T	T	C	A	A
54	A	A	A	T	T	T
58	T	T	T	A	A	A
69	T	T	T	A	A	A
72	A	A	A	G	G	G
81	T	T	T	A	A	A
91	G	G	G	A	A	A
93	T	T	T	C	C	C
106	A	A	A	G	G	G
111	G	G	G	C	T	T
129	C	C	C	T	T	T
138	T	T	T	C	C	C
156	C	C	C	C	A	A
159	G	G	G	A	A	A
358	C	C	T	C	C	C
375	A	C	C	A	A	A
453	T	T	C	T	C	C
489	G	T	T	G	G	G
492	T	C	C	T	T	T
498	C	T	T	C	C	C
501	G	A	A	G	G	G
567	C	C	C	C	T	T
585	G	G	G	G	T	T
594	C	C	C	C	T	T
612	C	C	C	C	T	T
894	T	T	T	T	C	C
905	G	A	A	G	G	G
912	C	T	T	C	C	C
952	G	G	G	G	A	A
957	A	G	G	A	A	A
984	A	G	G	A	G	G
1131	C	T	T	C	C	C
